# The Effects of Fasting and Massive Diarrhea on Absorption of Enteral Vancomycin in Critically Ill Patients: A Retrospective Observational Study

**DOI:** 10.3389/fmed.2017.00070

**Published:** 2017-06-08

**Authors:** Takehiko Oami, Noriyuki Hattori, Yosuke Matsumura, Eizo Watanabe, Ryuzo Abe, Taku Oshima, Waka Takahashi, Shingo Yamazaki, Tatsuya Suzuki, Shigeto Oda

**Affiliations:** ^1^Department of Emergency and Critical Care Medicine, Chiba University Graduate School of Medicine, Chiba, Japan; ^2^Division of Pharmacy, Chiba University Hospital, Chiba, Japan

**Keywords:** vancomycin, enteral administration, therapeutic drug monitoring, *Clostridium difficile* infection, critical care

## Abstract

**Purpose:**

Although vancomycin (VCM) is not absorbed from healthy intestinal mucosa, elevations in the serum VCM concentrations have been reported in some cases. The aims of this study are to evaluate the necessity of therapeutic drug monitoring (TDM) during enteral VCM administration in critically ill patients.

**Materials and methods:**

In this retrospective study, we enrolled 19 patients admitted to our intensive care unit who were treated with enteral VCM from December 2006 to January 2014. Clinical factors were compared between two groups: Group E whose serum concentrations were detectable, and Group N whose concentrations were below the detection limit of the VCM assay.

**Results:**

Group E comprises 7 patients, and Group N comprises 12 patients. The fasting duration in Group E was significantly longer compared with that in Group N (17 vs. 8 days, *p* = 0.023). Furthermore, there was a significant correlation between the serum VCM concentrations and the fasting duration (*r* = 0.79, *p* < 0.0001), and the amount of diarrhea (*r* = 0.46, *p* = 0.046). No difference was observed in the amount of diarrhea at the time of TDM (Group E; 1,850 mL vs. Group N; 210 mL, *p* = 0.055) and in the Sequential Organ Failure Assessment subscore for the renal system at the time of TDM (Group E; 4.0 vs. Group N; 1.5, *p* = 0.068).

**Conclusion:**

Long durations of fasting and massive diarrhea were associated with elevations in the serum VCM concentrations, which suggested that TDM might be necessary during enteral VCM administration in critically ill patients.

**Trial registration:**

UMIN Clinical Trials Registry identifier UMIN000016955.

## Introduction

An oral or enteral administration of vancomycin (VCM) hydrochloride is mainly used for the treatment of a *Clostridium difficile* infection (CDI) (1). VCM is not absorbed through healthy intestinal mucosa (2–4); however, serum VCM concentrations were elevated due to renal dysfunction and intestinal mucosal damage with severe inflammation in the previous reports (5–8). As high concentrations of serum VCM lead to the adverse effects including renal dysfunction and ototoxicity (9, 10), therapeutic drug monitoring (TDM) is recommended in patients with renal failure during oral or enteral VCM administration (2). We previously reported a critically ill patient with severe colitis and renal insufficiency presented elevated serum VCM concentrations above toxic levels during enteral VCM administration (11). Although the risk factors for elevated serum VCM concentrations have been suggested in the case series study (12), there is scarce evidence regarding the critically ill patients during oral or enteral VCM administration.

We hypothesized that clinical course and the severity of the patients are associated with elevated serum VCM concentrations. The objective of this study is to elucidate the relationship between clinical factors and elevation of serum VCM concentrations in critically ill patients. We conducted a retrospective observational study enrolling patients who were administered oral or enteral VCM.

## Materials and Methods

### Patients

We selected patients who were admitted to the medical–surgical intensive care unit (ICU) (22 beds) of Chiba University Hospital (Chiba, Japan) between December 2006 and January 2014, who were administered oral or enteral VCM, and in whom serum VCM concentrations were measured. VCM was initiated for patients who were diagnosed or suspected according to their symptoms. Pediatric patients under the age of 18 and patients concomitantly administered parenteral VCM were excluded from the study. Patients in the primary cohort were divided into two groups according to their highest VCM concentration: Group E included patients whose blood concentrations were elevated above the lower limits of the quantification of the VCM assay, and Group N comprised patients with concentrations below the detection limit of the VCM assay.

### VCM Monitoring

Vancomycin hydrochloride^®^ (Kobayashi Kako Co., Ltd., Fukui, Japan) was administered enterally when a CDI was diagnosed or suspected. The attending physicians assessed the therapeutic dose, the timing of the serum VCM concentration measurements, and the necessity for the cessation or adjustment of the dose of VCM. ICU physicians and clinical pharmacists discussed the plan for TDM in daily ICU conferences. Serum VCM concentrations were measured as the total fraction in a fluorescence polarization immunoassay method with an AxSYM^®^ analyzer (Abbott Japan Co., Ltd., Tokyo, Japan) until December 2009. The lower and upper limits of the quantifications of the assay were 2.0 and 100.0 µg/mL, respectively. In January 2010, the above method was replaced with a chemiluminescent immunoassay method with an ARCHITECT i1000SR^®^ analyzer (Abbott Japan Co., Ltd.). The lower and upper limits of the quantifications of the new assay were 0.24 and 100.0 µg/mL, respectively. The differences in the quantifications that were performed by the two assays were negligible.

### Data Collection

For the VCM treatment data, the indications of enteral VCM administration, the daily dose, the cumulative dose, the duration of administration, and the renal toxicity as an adverse effect were recorded. The renal toxicity was defined as either a 0.5 mg/dL increase from baseline in serum creatinine (SCr) or a ≥50% increase from baseline in SCr based on serial SCr measurements during enteral VCM administration (13). The demographic data that were obtained included age, gender, admission diagnosis, Acute Physiology and Chronic Health Evaluation II score (14), and Sequential Organ Failure Assessment (SOFA) score (15) on ICU admission. The length of the ICU stay and ICU mortality were also recorded. The clinical course data at the time of TDM were recorded as follows: SOFA score, gastrointestinal symptoms (amount of diarrhea or melena), fasting duration, and renal replacement therapy. Diarrhea was defined as loose or liquid stools that occurred three or more times per day with a stool weight greater than 200–250 g/day (or greater than 250 mL/day) (16).

### Statistical Analysis

The categorical data are presented as absolute numbers and percentages and were analyzed with Fisher’s exact tests or Chi-square test as appropriate. Also, the continuous data are presented as median and quartile and were analyzed with Mann–Whitney *U*-tests. Spearman correlation coefficients were also calculated. The statistical analyses were performed using GraphPad Prism 6 (GraphPad Software, San Diego, CA, USA). Differences were considered significant at *p* < 0.05 (two-tailed test).

## Results

**Figure 1:**
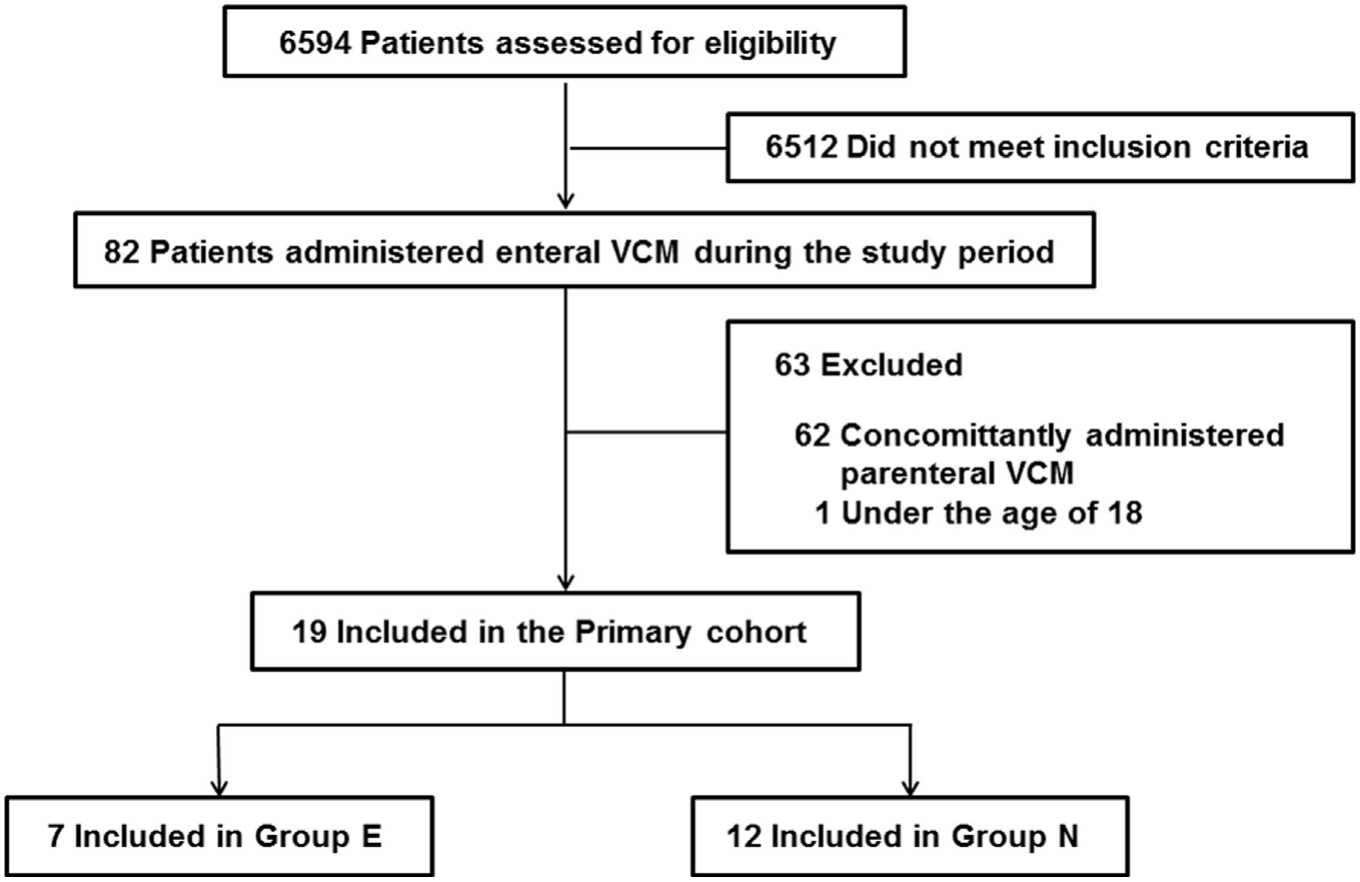
The flowchart of the study enrollment.

During the study period, 6,594 patients were admitted to the ICU, and 82 patients were administered oral or enteral VCM. Patients under the age of 18 and patients who were concomitantly administered parenteral VCM were excluded. Thus, we enrolled 19 patients. Seven patients were in Group E, and 12 were in Group N (Figure 1, Online Resource 1). No significant differences were observed in age, sex, admission diagnosis, the length of ICU stay, and ICU mortality between the two groups (Table 1).

**Table 1:** Patients characteristics.

	Group E (*n* = 7)	Group N (*n* = 12)	*p-*Value
Age, year	65 (56.5–79.0)	64 (55.0–77.2)	0.98
Male, *n* (%)	4 (57.1)	6 (50.0)	1.0
APACHE II score on ICU admission	32 (26.5–37.5)	25 (18.7–30.0)	0.13
SOFA score on ICU admission	12 (8–14)	9 (5.5–12.2)	0.32
Respiratory system	3 (1.0–3.0)	2.5 (1.0–3.0)	0.65
Coagulation system	2 (0–3.0)	2 (1.0–3.7)	0.70
Liver system	2 (1.0–2.0)	1 (0–2.0)	0.50
Cardiovascular system	1 (0–4.0)	2 (1.2–3.0)	0.71
Nervous system	1 (1.0–3.0)	1 (0.2–2.0)	0.62
Renal system	2 (2.0–4.0)	2 (2.0–3.7)	0.54
Admission diagnosis, *n* (%)			0.63
Infection	3 (42.8)	8 (66.7)	
Pneumonia	1 (33.3)	0 (0)	
Intraabdominal infection	2 (66.6)	6 (75.0)	
Skin or soft-tissue infection	0 (0)	1 (12.5)	
Other	0 (0)	1 (12.5)	
Gastrointestinal disorder	2 (28.5)	2 (16.7)	
Hematologic disorder	1 (14.2)	1 (8.3)	
Others	1 (14.2)	1 (8.3)	
Length of ICU stay (days)	36 (15–47)	20 (10–26)	0.099
ICU mortality, *n* (%)	3 (42.8)	3 (25.0)	0.61

*Data are expressed as median (interquartile range), or number (%), as appropriate*.

*APACHE II, Acute Physiology and Chronic Health Evaluation II; SOFA, Sequential Organ Failure Assessment; ICU, intensive care unit*.

### VCM Treatment and Serum VCM Concentration

No significant difference in the indications for VCM administration between the groups was found, and the most common VCM dose was 2 g/day. The median serum VCM concentration in Group E was 16.4 µg/mL (interquartile range, 5.1–27.0). No significant differences were observed in the daily dose, dose interval, cumulative VCM dose, and time from treatment initiation to TDM (Table 2).

**Table 2:** VCM treatment and serum VCM concentration.

	Group E (*n* = 7)	Group N (*n* = 12)	*p*-Value
**Indication**			0.63
CDI	2 (28.5)	6 (50.0)	
Empiric therapy	5 (57.1)	6 (25.0)	
**Daily dose (g/day)**			0.17
0.5	1 (14.2)	0 (0)	
1.5	0 (0)	3 (25.0)	
2	6 (85.7)	9 (75.0)	
**Dose interval (h)**			0.26
6	7 (100)	9 (75.0)	
8	0 (0)	3 (25.0)	
**Cumulative dose (g)**	16 (8.0–23)	9.5 (3.7–18)	0.30
**Route of administration**			1.0
Oral or *via* gastric tube	7 (100)	12 (100)	
Rectal	0 (0)	0 (0)	
**Time from treatment initiation to TDM (day)**	9 (5–15)	5.5 (3–12)	0.24
**VCM concentration (μg/mL)**	16.4 (5.1–27.0)	–	–

*CDI, Clostridium difficile infection; VCM, vancomycin; TDM, therapeutic drug monitoring*.

### Clinical Course at the Time of TDM

**Figure 2:**
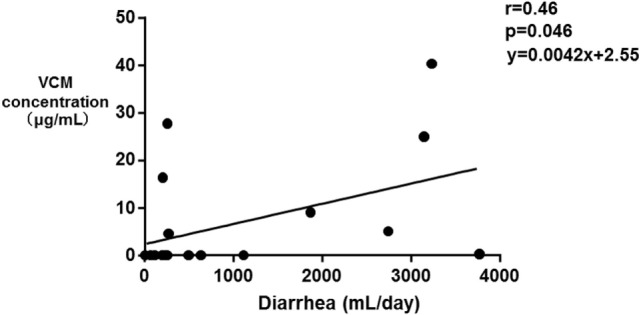
Correlation between the serum vancomycin (VCM) concentrations and the amount of diarrhea. Each plot depicts a relationship between the amount of diarrhea (milliliter per day) on the *x*-axis and the serum VCM concentration (microgram per milliliter) on the *y*-axis. A significant correlation between the serum VCM concentrations and the amount of diarrhea was observed (*r* = 0.46, *p* = 0.046).

**Figure 3:**
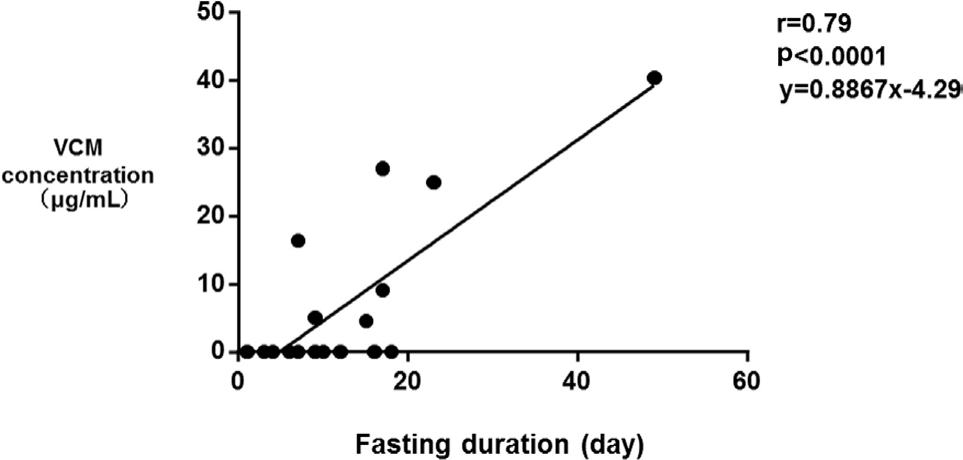
Correlation between the serum vancomycin (VCM) concentrations and the fasting duration. Each plot depicts a relationship between the duration of fasting (day) on the *x*-axis and the serum VCM concentration (microgram per milliliter) on the *y*-axis. There was also a significant correlation between the serum VCM concentrations and the fasting duration (*r* = 0.79, *p* < 0.0001).

The median SOFA scores at the time of TDM in Groups E and N were 12 and 9, respectively (*p* = 0.39). No significant differences were observed in the SOFA subscore for the renal system (*p* = 0.068), proportions of patients improving renal function from ICU admission to the time of TDM (*p* = 0.058), and the amount of diarrhea at the time of TDM (*p* = 0.055). The fasting duration in Group E was significantly longer compared with that in Group N (*p* = 0.023, Table 3). Among these three variables, there was a significant correlation between the serum VCM concentrations and the fasting duration (*r* = 0.79, *p* < 0.0001) and the amount of diarrhea (*r* = 0.46, *p* = 0.046) (Figures 2 and 3). The reasons we delayed enteral feeding included melena, hemodynamic instability, and abdominal compartment syndrome accompanying acute pancreatitis. The rate of enteral feeding in the Group E was lower than that in the Group N (Group E vs. N, 28.5 vs. 50%) (Table 3).

**Table 3:** Clinical course at the time of therapeutic drug monitoring (TDM).

	Group E (*n* = 7)	Group N (*n* = 12)	*p*-Value
SOFA score	12 (8.0–14.0)	9 (5.5–13.7)	0.39
Respiratory system	2 (1.0–3.0)	1.5 (1.0–2.7)	0.40
Coagulation system	2 (0–3.0)	2.5 (0.2–4.0)	0.69
Liver system	2 (1.0–3.0)	2 (0.2–2.7)	0.65
Cardiovascular system	1 (0–3.0)	0.5 (0–2.0)	0.89
Nervous system	1 (0–2.0)	1 (0–2.5)	0.97
Renal system	4 (2.0–4.0)	1.5 (0–3.7)	0.068
Comparison of renal SOFA score on ICU admission (1) with at the time of TDM (2)			0.058
(1) > (2)	1 (14.2)	7 (58.3)	
(1) = (2)	4 (57.1)	5 (41.6)	
(1) < (2)	2 (28.5)	0 (0)	
Gastrointestinal symptom			
Amount of diarrhea (mL/day)	1,850 (245–3,140)	210 (15–595)	0.055
Melena, *n* (%)	3 (42.8)	1 (8.3)	0.11
Fasting duration (days)	17 (12–20)	8 (4–10.5)	0.023
Enteral feeding	2 (28.5)	6 (50.0)	0.63
RRT, *n* (%)	5 (71.4)	7 (58.3)	0.65

*Data are expressed as median (interquartile range), or number (%), as appropriate*.

*SOFA, Sequential Organ Failure Assessment; RRT, renal replacement therapy; ICU, intensive care unit*.

## Discussion

This study was conducted to identify the risk factors associated with elevated serum VCM concentrations during enteral administration in the critically ill patients. The results showed that the fasting duration and the amount of diarrhea were associated with elevation of serum VCM concentrations. Also, there was a trend toward higher SOFA subscore for the kidney system in Group E at the time of TDM without a significant difference.

It is assumed that VCM is not absorbed through the intestinal mucosa because it has a large molecular weight and is hydrophilic. Therefore, serum VCM concentrations are rarely elevated in patients with normal intestinal mucosa and renal function (17). Conversely, an increase of drug penetration and drug accumulation due to lower renal excretion leads to elevated serum VCM concentrations during enteral VCM administration.

We speculated that the penetration of VCM is related to the severity of the intestinal mucosal damage. Although elevations of serum VCM concentrations are not observed in many cases with pseudomembranous colitis (18), it has been reported in a few cases with severe intestinal lesions or other intestinal disorders, including a case with graft vs. host disease accompanying pseudomembranous colitis (19). In our study, massive diarrhea was associated with elevated serum VCM concentrations. In previous studies, the highest serum VCM concentration, 58.7 µg/mL, with an estimated penetration rate into the blood of 16.8%, was reported in a patient with active CDI-induced pseudomembranous colitis (20). The highest concentration in our study was 40.4 µg/mL, with a penetration rate into the blood of 33%, in a patient with severe pseudomembranous inflammation of almost all the intestinal mucosa (11). These findings suggested that the severity of the intestinal mucosal damage contributed to the elevations in the serum VCM concentrations. In addition, fasting is related to the intestinal mucosal impairment (21), which is consistent with the results of our study that the fasting duration was associated with the serum VCM concentration elevations. Gut atrophy, the loss of the gut barrier, and increased gut permeability after inflammatory insults have been documented to be associated with a long duration of fasting.

Furthermore, a gastrointestinal function is often impaired in critically ill patients because the intestinal mucosa is structurally and functionally damaged (22–24). Therefore, this impairment might cause drug penetration through the intestinal mucosa. The plausibility of this hypothesis is supported by the report that ICU admission was a risk factor for serum VCM concentration elevations during enteral administration (12). In addition, tobramycin was detected in the blood during enteral tobramycin administration that was a component of the selective decontamination of the digestive tract in critically ill patients (25, 26).

While renal impairment has been reported to be a risk factor for elevated serum VCM concentrations during enteral or oral administration in some cases (6, 7), it has also been reported that a patient with normal renal function developed significant absorption of oral VCM (5). Theoretically, renal impairment should be a risk factor in elevation of serum VCM concentration. However, the lack of a significant association between the renal SOFA subscore and the serum VCM concentrations during enteral VCM administration in this study suggested that increased drug penetration contributed to the elevations of serum VCM concentrations rather than drug accumulation due to lower renal excretion. Although it is impossible to judge retrospectively whether the cause of renal failure was the adverse event due to the elevation of serum VCM concentration or severer pathology, the causal relationship between the serum VCM elevation and renal dysfunction could not be entirely excluded. Therefore, TDM might be important to avoid the toxic range of VCM concentrations in spite of the difficulty in predicting elevation of serum VCM concentrations according to renal function.

This study had several limitations. First, it was a single-center study, and the sample size was small. Second, selection bias could not be denied and causal relationship could not be shown in this study population because this study relied on retrospective observational data. Third, the details of the pharmacokinetics could not be examined because no definitive rules were applied to the timing and number of serum concentration measurements. Fourth, our institution did not have a specific protocol regarding the VCM dose and the timing of TDM. Due to these limitations, further prospective studies should be performed to elucidate the risk factors that are related to elevated serum VCM concentrations and the pharmacokinetics following enteral administration.

In conclusion, long durations of fasting and massive diarrhea were associated with elevations in the serum VCM concentrations, suggesting that TDM might be necessary during enteral VCM administration in critically ill patients. Further research clarifying the risk factors of elevated serum VCM concentrations during enteral administration is warranted.

## Ethics Statement

This study was conducted following standards of Good Clinical Practice Guidelines and principle of the Declaration of Helsinki. Ethical approval was obtained from the institutional review board of Chiba University. At the time of admission to the hospital, all adult patients and the parents/legal guardians of all non-adult patients provided written informed consent for their data to be used in future research. The study was registered at University Hospital Medical Information Network (UMIN) clinical trials registry (ID: UMIN000016955).

## Author Contributions

TO planned the study and collected patient data, performed the statistical analysis, and drafted the manuscript. NH helped to draft the manuscript. YM helped to draft the manuscript and perform the statistical analysis. EW, RA, TO, and WT participated in the data collection. SY and TS performed measurement of the VCM assay and advised to draft the manuscript. SO critically revised the manuscript for important intellectual content. All the authors read and approved the final manuscript.

## Conflict of Interest Statement

The authors declare that the research was conducted in the absence of any commercial or financial relationships that could be construed as a potential conflict of interest.

## Abbreviations

VCM, vancomycin; CDI, *Clostridium difficile* infection; TDM, therapeutic drug monitoring; ICU, intensive care unit; APACHE II, Acute Physiology and Chronic Health Evaluation II; SOFA, Sequential Organ Failure Assessment; IQR, interquartile range.
