# Supplementary material for: The Effects of Fasting and Massive Diarrhea on Absorption of Enteral Vancomycin in Critically Ill Patients: A Retrospective Observational Study
Source: Front Med (Lausanne). 2017 Jun 8;4:70. doi: 10.3389/fmed.2017.00070 (PMC5462912; doi:10.3389/fmed.2017.00070)
Supplement: Supplementary file 1 [file table_1.docx]

**Table E1 ( Online resource 1 ) Group E patient profiles**

| Patient | Admission diagnosis | SOFA score † | Renal dysfunction | Blood purification | GI symptoms | Serum VCM concentration  (μg/mL) | Time from treatment initiation to measurement (d) |
| --- | --- | --- | --- | --- | --- | --- | --- |
| 1 | Acute pancreatitis | 13 | ＋ | CRRT | Melena | 40.4 | 28 |
| 2 | Respiratory failure | 8 | － | ― | Diarrhea | 4.6 | 5 |
| 3 | Enteritis | 18 | ＋ | ― | Melena | 27.0 | 9 |
| 4 | Leukemia | 14 | ＋ | CRRT | Melena | 9.1 | 9 |
| 5 | Colon cancer | 12 | ＋ | CRRT | Diarrhea | 25.0 | 10 |
| 6 | Pneumonia | 4 | ＋ | HD | Diarrhea | 5.1 | 15 |
| 7 | Acute pancreatitis | 12 | － | ― | Diarrhea | 16.4 | 3 |

SOFA, Sequential Organ Failure Assessment; CRRT, continuous renal replacement therapy; HD, hemodialysis; GI, gastrointestinal; VCM, vancomycin

**†At the time of therapeutic drug monitoring**
